# Donor-derived cell-free DNA is a valuable monitoring tool after single lung transplantation: Multicenter analysis

**DOI:** 10.1016/j.jhlto.2024.100155

**Published:** 2024-08-28

**Authors:** Ambalavanan Arunachalam, Fatima Anjum, Justin P. Rosenheck, Reinaldo Rampolla, Reda Girgis, Howard J. Huang, Kathryn Crabtree, Sarah McCormick, Zhiji Zhang, Sangeeta Bhorade, David J. Ross

**Affiliations:** a Division of Pulmonary and Critical Care, Northwestern University Feinberg School of Medicine, Chicago, Illinois; b Lewis Katz School of Medicine, Temple University, Philadelphia, Pennsylvania; c Division of Pulmonary, Critical Care, and Sleep Medicine, The Ohio State University, Columbus, Ohio; d Cedars-Sinai Medical Center, Department of Cardiac Surgery, Los Angeles, California; e Corewell HealthCare and Michigan State University College of Human Medicine, Grand Rapids, Michigan; f Houston Methodist Lung Transplant Center, Houston Methodist Hospital, Houston, Texas; g Natera, Inc., Austin, Texas

**Keywords:** cell-free DNA, lung transplant, allograft rejection, single lung transplant, biomarker

## Abstract

**Background:**

Donor-derived cell-free DNA (dd-cfDNA) is a nonspecific plasma biomarker for tissue injury that has been validated for monitoring acute rejection (AR) after lung transplantation (LT). However, no studies to date have focused specifically on single lung transplantation (SLT). Herein, we report the performance of dd-cfDNA in detecting AR in SLT from 6 academic centers that implemented this biomarker surveillance in their standard of practice (SOP).

**Methods:**

dd-cfDNA test results were corrected for SLT by an algorithm in the Clinical Laboratory Improvement Amendments (CLIA) laboratory to permit comparison against the same 1.0% threshold used in double-lung transplant. Investigators reviewed patient SOP electronic medical record clinical data to assign test results into cohorts based on clinical allograft health status. To avoid ambiguity in interpretation, samples drawn after a prior AR or infection event or without histopathologic confirmation of AR were excluded from further analysis. Diagnostic cohorts included AR (N=25 samples), healthy (STABLE, N=137), allograft infection (INFXN, N=41), chronic lung allograft dysfunction (CLAD, N=7), and “OTHER” types of graft injury (N=12).

**Results:**

The study included a total of 257 dd-cfDNA results from 103 SLT patients with one patient excluded due to active cancer. Samples were drawn a median of 233 days (interquartile range: 96-489) after SLT. Laterality for SLT (R vs L) and median dd-cfDNA fraction in AR and STABLE cohort were not statistically different. The median dd-cfDNA fraction was elevated with AR (1.8%) and INFXN (1.1%) vs STABLE (0.46%; *p* < 0.0001). dd-cfDNA with CLAD was also significantly higher than STABLE cohort (*p* = 0.0155). The area under receiver operator characteristics curve was 0.850 (95% confidence interval: 0.72-0.95, *p* < 0.0001) for AR vs STABLE cohort. Applying the dd-cfDNA threshold ≥1.0% for detection of AR yielded a sensitivity = 77.8%, specificity = 84.6%, positive predictive value = 38.31%, and negative predictive value = 96.83%.

**Conclusions:**

These multicenter data, incorporating real-world experiences, support the clinical validity and utility of dd-cfDNA monitoring of SLT recipients. Additional studies of the impact of biomarker surveillance on clinically meaningful outcomes should be forthcoming from robust, prospective, and clinical trials already in progress.

## Background

Donor-derived cell-free DNA (dd-cfDNA) is a nonspecific plasma biomarker for allograft injury that has been validated to detect acute rejection (AR) after lung transplantation (LT). However, despite representing a more vulnerable, higher risk patient demographic with greater associated risks of protocol transbronchial biopsy (TBBx) procedures, there is only limited published data addressing the use of noninvasive plasma dd-cfDNA monitoring in single lung transplantation (SLT). As evidenced by Organ Procurement and Transplantation Network (OPTN)/ Scientific Registry of Transplant Recipients (SRTR) data, as of 2021, of the 2,569 LT procedures performed per annum, approximately 20% represent SLT procedures.^1^ Despite better quality-of-life metrics and 5-year allograft survival rates with double-lung vis-à-vis SLT (International Society for Heart and Lung Transplantation [ISHLT] data: 59% vs 48%), the decision to perform SLT is typically made based on recipient characteristics—advanced age, comorbidities, frailty, type of native lung disease, body morphology, and projected donor organ availability.^2^, ^3^ Therefore, the SLT cohort, with potentially inferior outcomes and an increased risk for serious adverse events resulting from invasive TBBx procedures, may be expected to benefit most from a noninvasive surveillance biomarker solution. The aim of our study was to analyze the performance characteristics of plasma dd-cfDNA specifically from SLT patients in real-world experience with a clinically available assay at centers providing routine standard of practice (SOP) care. We hypothesized that the dd-cfDNA test performance in detecting AR in this SLT cohort is comparable to double-lung experiences when incorporating a correction factor (multiplied by 2), thereby accounting for the difference in total lung mass.

## Methods

### Study design/dataset development

The study was approved (IRB 20-049-ALL) as a Clinical Laboratory (Natera, Inc.; Austin, TX) Improvement Amendments (CLIA) quality initiative. dd-cfDNA testing (the Prospera^1^ Lung test) was performed as part of routine clinical care for SLT patients at 6 LT centers, and test results were analyzed by each investigator in combination with the patients' SOP clinical data. Electronic medical records (EMRs) were retrospectively analyzed for relevant clinical events occurring ±6 weeks of the dd-cfDNA test date. Since biopsy-related surgical trauma and management changes/treatments driven by biopsy results can affect dd-cfDNA levels,^4^ samples were excluded if they were drawn immediately after a biopsy/invasive allograft procedure (regardless of histopathologic result) or up to 6 weeks after a confirmed AR. Samples drawn up to 6 weeks after an identified infection were excluded to mitigate the effects of the treatment on dd-cfDNA. Samples matched with a suspected but not histopathologic-confirmed AR were excluded. The definitions used for classification are delineated below.

The study has been performed in full adherence to the Declaration of Helsinki. The clinical and research activities reported here are consistent with the Principles of the Declaration of Istanbul as outlined in the Declaration of Istanbul on Organ Trafficking and Transplant Tourism.

### Donor-derived cell-free DNA analysis

Blood samples were collected in two 10-ml cell-free DNA (cfDNA) BCT tubes (Streck, Omaha, NE) that contain preservative for the inhibition of nucleases and cellular degradation, thereby maintaining cfDNA stability for up to 14 days at ambient temperature. Tubes were provided to physicians as part of the test kit. The Prospera Lung test was performed in the Natera CLIA-certified, College of American Pathologists (CAP)-accredited laboratory in San Carlos, California. cfDNA was isolated from the plasma and amplified using a massively multiplexed polymerase chain reaction assay targeting a curated panel of more than 13,000 single-nucleotide polymorphisms designed to maximize variant frequency across ethnicities.^5^ For each sample, amplicons were sequenced by next-generation sequencing, performed on the Illumina NextSeq500 on rapid run with an average of 14 to 15 million reads per sample, and sequencing data processed to estimate the fraction of dd-cfDNA (expressed as a percentage) in relation to total cfDNA. The dd-cfDNA raw result was then corrected for the SLT cohort (2×-multiplied) in the CLIA-lab automated algorithm (Natera, Austin, TX) to allow comparison of reported test result against the same threshold adopted for double lung of ≥1.0%. dd-cfDNA test results are typically returned to the provider from the CLIA-certified lab within 48 to 72 hours from the time of sample receipt.

### Cohort development

Each dd-cfDNA test included in the analysis was deidentified and assigned to a “diagnostic cohort” based on the predominant organ health status—AR, healthy (STABLE), allograft infection (INFXN), chronic lung allograft dysfunction (CLAD), or “other” conditions potentially associated with allograft dysfunction (OTHER). Patient allograft status at the time of each dd-cfDNA test was retrospectively determined based on histopathology (TBBx) results along with EMR review of pertinent clinical progress notes, microbiology, human leukocyte antigen (HLA) donor-specific antibodies class I and class II, spirometry, and treatment that occurred proximal to the biopsy. The AR cohort included acute cellular rejection (ACR) defined as ISHLT grade A1 to A4 in addition to episodes of “treated” antibody-mediated rejection (AMR) according to ISHLT criteria.^6^, ^7^ Histology consistent with ACR or AMR was provided for all AR cases; in cases of AMR, histology was supportive of diagnosis, with other criteria needed for diagnosis, per ISHLT guidelines. The CLAD cohort was categorized by ISHLT accepted criteria inclusive of either obstructive, restrictive, or mixed phenotypes.^8^ The INFXN cohort was defined according to ISHLT criteria by EMR interrogation and had received antibiotic therapy.^9^ The STABLE cohort was determined by the investigator based on an absence of clinical and/or histopathologic evidence of AR, INFXN, respiratory symptoms, or spirometry decrement.

### Statistical analysis

The distribution of dd-cfDNA in each cohort was analyzed and depicted as median (25%-75% interquartile range [IQR]) and compared to STABLE by nonparametric statistical analysis (Mann-Whitney U test, 2-sided). Adjustments for the type I error rate due to multiple comparisons were made using the false discovery rate (FDR) method (Benjamini/Hochberg).

For all test performance estimations, a subsampling approach was used to account for potential dependence between samples from the same patient, implementing random selection within each patient to ensure selected samples meet the independent criteria as samples were more than 90 days apart or sample diagnoses were different (i.e., AR vs STABLE). To better assess the variability of test performance in various sample pools, additional bootstrapping was performed with replacement among samples selected from the above subsampling step. The subsampling and bootstrapping were repeated for 10,000 iterations. For the binary distinction of AR vs STABLE, area under receiver operator characteristics curve (AUROC, Wilson/Brown) and the following test performance metrics were calculated in each iteration using a dd-cfDNA threshold ≥1%: sensitivity, specificity, positive predictive value (PPV), negative predictive value (NPV) (implementing the study cohort prevalence), positive likelihood (+LR), and negative likelihood (−LR) ratio. The point estimate and 95% confidence interval (95% CI) were the median, 2.5th percentile, and 97.5th percentile from the 10,000 results.

Linear regression was applied for dd-cfDNA fraction vs time post-SLT for the STABLE test results to assess for differences related to early vs late epochs after transplantation.

## Results

### Data overview

**Figure 1:**
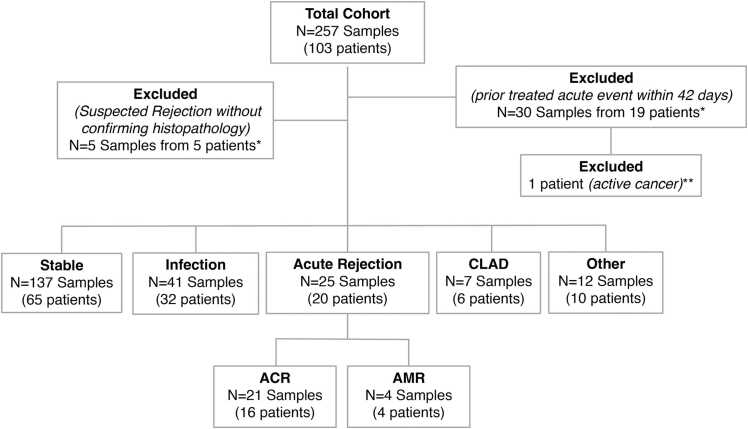
Cohort assignments and exclusions for all samples and patients retrospectively assessed for this study. Cohorts included STABLE (healthy allograft); AR (acute rejection including both acute cellular rejection [ACR] and antibody-mediated rejection [AMR]); INFXN (allograft infection); CLAD (chronic lung allograft dysfunction); OTHER (other causes of graft dysfunction, not described). Different samples from the same patient may be categorized into different cohorts or excluded based on criteria (see Methods). As such, patients may be counted in more than 1 category. *Patients with excluded samples had additional samples that met inclusion criteria. **All samples from this patient met exclusion criteria and as such, the entire patient was excluded from further analysis.

Between December 10, 2021 and October 27, 2023, a total of 257 plasma dd-cfDNA test results with available associated EMR clinical data from 103 SLT patients at 6 academic LT centers were reviewed. Figure 1 depicts the total samples and patients assigned to each diagnostic cohort or excluded from further analysis. A total of 30 samples were excluded from further analysis due to treatment of a previously assigned clinical event (infection or rejection) in the 42 days before dd-cfDNA testing, including all samples from 1 patient with metastatic cancer. Five samples were excluded from analysis for clinically suspected AR but without histopathologic confirmation. Demographics of the remaining 102 patients demonstrated a median patient age at the time of SLT of 68.8 years (IQR: 64.1-71.5) with a sex distribution (F:M) of 0.65. Laterality for SLT was designated 52.9% for left, 44.1% for right, and 2.9% unreported. Median time post-SLT until the initial dd-cfDNA lab draw (all cohorts combined) was 233 days (IQR: 96-489).

### dd-cfDNA was elevated in the allograft injury cohorts of AR, INFXN, and CLAD, compared to a STABLE cohort

**Figure 2:**
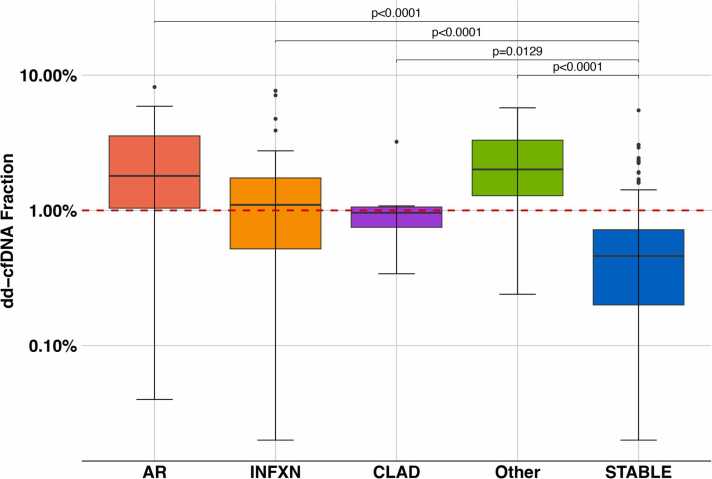
Box and whisker plots for dd-cfDNA levels in cohorts after SLT. Cohorts included STABLE, healthy allograft; AR, acute rejection including both acute cellular rejection and antibody-mediated rejection; INFXN, allograft infection; CLAD, chronic lung allograft dysfunction; OTHER (other causes of graft dysfunction). Box = 25%-75% interquartile range, horizontal line = median dd-cfDNA value, whiskers = 95% confidence interval, dots = outlier values. *p*-values FDR-adjusted. dd-cfDNA, donor-derived cell-free DNA; FDR, false discovery rate; SLT, single lung transplantation.

For the AR cohort (N = 25 samples), there were N = 21 dd-cfDNA tests associated with ACR, including ISHLT grade A1 (N = 8), A2 (N = 5), A3 (N = 0), A4 (N = 1), and unspecified (N = 7). There were 4 dd-cfDNA tests associated with ISHLT probable/definite acute AMR. Median dd-cfDNA fraction for AR cohort (1.80%; IQR:1.04%-3.56%) was significantly elevated compared to STABLE (0.46%; 0.20%-0.72%; *p* < 0.0001) (Table 1, Figure 2). Although dd-cfDNA fraction was elevated in all 4 AMR samples (8.18%, 5.90%, 2.32%, and 4.72%) due to the small N, we did not analyze further as a distinct diagnostic cohort. Further, the median dd-cfDNA was significantly elevated with graft-associated INFXN (1.10%; 0.52%-1.74%) compared to STABLE (*p* < 0.0001). Prior studies have reported variability in dd-cfDNA fractions associated with CLAD.^10^, ^11^, ^12^, ^13^ As an exploratory assessment, we analyzed dd-cfDNA in samples associated with established CLAD diagnosis in our SLT cohort. Among 7 CLAD samples, the median dd-cfDNA was significantly elevated (0.96%; 0.75%-1.06%) compared to STABLE (*p* = 0.0155). Representing a mixed cohort with a spectrum of causes of potential graft injury, the OTHER cohort (allograft complication related to bronchial anastomosis, pleural disease, and pulmonary embolism) was also associated with significant dd-cfDNA elevations (N = 12; 2.01%; 1.28%-3.31%; *p* < 0.0001).

**Table 1:** Donor-Derived Cell-Free DNA (dd-cfDNA) Fractions for Cohorts

Cohort	N	Median (IQR)	*p*-value^a^	FDR-adjusted *p*-value^b^
STABLE	137	0.46% (0.20-0.72)		
AR	25	1.80% (1.04-3.56)	<0.0001	<0.0001
INFXN	41	1.10% (0.52-1.74)	<0.0001	<0.0001
CLAD	7	0.96% (0.75-1.06)	0.0129	0.0155
OTHER	12	2.01% (1.28-3.31)	<0.0001	0.0001

Abbreviations: AR, acute rejection; CLAD, chronic lung allograft dysfunction; FDR, false discovery rate; INFXN, infection; IQR, interquartile range; OTHER (other causes of graft dysfunction, refer to text).

Mann-Whitney U test with FDR-adjusted *p-values compared to STABLE (healthy) for diagnostic cohorts.*

a Mann-Whitney compared to STABLE cohort.

b Mann-Whitney U test with FDR-adjusted p-values compared to STABLE cohort.

**Figure 3:**
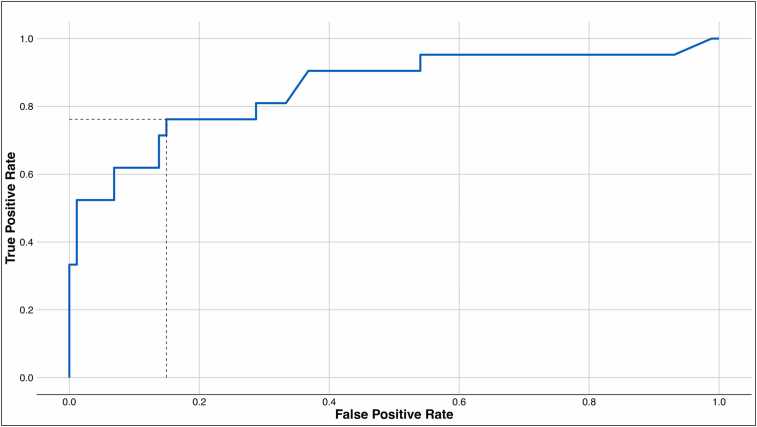
Receiver operator characteristics (ROC) curve for AR vs STABLE cohorts, representing median curve by bootstrapping 10,000 iterations; area under curve (AUC) = 0.85 (95% CI: 0.72-0.95). Dashed lines represent true and false positive rates at the 1.0% threshold for dd-cfDNA. CI, confidence interval; dd-cfDNA, donor-derived cell-free DNA; STABLE, healthy allograft.

The AUROC was 0.85 (0.72-0.95) for AR vs STABLE (Figure 3). Applying a dd-cfDNA threshold of 1.0% yielded the following performance metrics: sensitivity = 77.8% (95% CI: 58.33-94.12), specificity = 84.62% (76.00-91.95, PPV = 38.31% (26.94-55.03), NPV = 96.83% (94.8-99.16), +LR = 5.02 (2.98-9.89), and −LR = 0.26 (0.07-0.50). The prevalence of AR in this study was determined as 11.01%.

### STABLE cohort median dd-cfDNA fraction was unchanged over time post-SLT

To assess the potential for time as a covariate that may affect dd-cfDNA fraction post-transplantation, linear regression was performed and suggested no significant relationship of dd-cfDNA fraction vs time during the timeframe of dd-cfDNA testing with an R^2^ = 0.00268 (*p* = 0.538).

### Laterality of SLT (right vs left) demonstrated similar fractions for plasma dd-cfDNA in diagnostic cohorts

In Table 1, analyzing all cohorts collectively, median dd-cfDNA was not different for left (0.62%; IQR: 0.32-1.36) vs right (0.58%; 0.26%-1.46%) side allografts (*p* = 0.821). In the STABLE cohort, dd-cfDNA fraction was not different in the left (N = 68; 0.46%; 0.22%-0.73%) vs right side allografts (N = 61; 0.44%; 0.20%-0.84%; *p* = 0.821). Among the AR cohort, the left allograft demonstrated a nonsignificant higher median dd-cfDNA (N = 10; 2.43%; 0.91%-3.88%) relative to right side allografts (N = 15; 1.58%; 1.08%-2.80%) (*p* = 0.821) (Table 2).

**Table 2:** Analyzed Cohorts for Left vs Right Side Allografts for Donor-Derived Cell-Free DNA (dd-cfDNA) Fraction

	Left lung		Right lung			
Cohort	N of samples	Median (IQR)	N of samples	Median (IQR)	*p*-value^a^	FDR-adjusted *p*-value^b^
Overall	112	0.62% (0.32-1.36)	106	0.58% (0.26-1.46)	0.5889	0.8208
STABLE	68	0.46% (0.22-0.73)	61	0.44% (0.20-0.84)	0.8208	0.8208
AR	10	2.43% (0.91- 3.88)	15	1.58% (1.08-2.80)	0.5981	0.8208
INFXN	25	1.24% (0.72-1.74)	16	0.85% (0.32-1.92)	0.3850	0.8208
CLAD	3	0.96% (0.85-2.09)	4	0.90% (0.66-1.05)	0.7237	0.8208
OTHER	5	1.72% (1.18-2.26)	6	2.16% (1.43-4.81)	0.4102	0.8208

Abbreviations: AR, acute rejection; CLAD, chronic lung allograft dysfunction; FDR, false discovery rate; INFXN, allograft infection; IQR, interquartile range; OTHER, other causes of graft dysfunction (refer text); STABLE, healthy allograft.

a Mann-Whitney U test compared to STABLE cohort.

b Mann-Whitney U test with FDR-adjusted p-values compared to STABLE cohort.

### Time post-SLT was longer for AR and CLAD but not for INFXN vs the STABLE cohort

The median time dd-cfDNA was tested post-SLT was 230.0 days (130-334) in the STABLE cohort, which was significantly earlier than in the AR cohort (456.0 days; 153-570, FDR-adjusted *p* = 0.046) and CLAD (720.0 days (419.5-1617.0, *p* < 0.0001) but not for INFXN (264.0 days; 129-503, *p* = 0.266). Time post-transplant for OTHER (*p* = 0.38) cohort was not different from STABLE. There was an insignificant trend for the CLAD cohort (671.0 days; 473-825; FDR-adjusted *p* = 0.1435) as later compared to AR events (Table 3).

**Table 3:** Donor-Derived Cell-Free DNA (dd-cfDNA) Test Performance Characteristics for Acute Rejection (AR) vs STABLE Cohorts

Characteristic	AR vs STABLE (95% CI)
Sensitivity	77.78% (58.33-94.12)
Specificity	84.62% (76.00-91.95)
PPV	38.31% (26.94-55.03)
NPV	96.83% (94.18-99.16)
+LR	5.02 (2.98-9.89)
−LR	0.26 (0.07-0.50)
AUC-ROC	0.85 (0.72-0.95)

Abbreviations: +LR, positive likelihood ratio; −LR, negative likelihood ratio; AUC-ROC, area under curve of receiver operator characteristics; CI, confidence interval; NPV, negative predictive value; PPV, positive predictive value.

dd-cfDNA threshold ≥1.0% was used for determination of test performance characteristics. Study prevalence for AR was 11%.

## Discussion

LT represents a viable source of hope for more than 4,000 patients suffering from a spectrum of causes of end-stage lung disease in the United States and who currently are “wait listed” with United Network for Organ Sharing (UNOS).^1^ Despite the witnessed improvement in surgical outcomes, according to data reported by ISHLT, the median unadjusted adult lung allograft survival for the most recent era remains only 6.3 years—7.3 years for double lung and only 4.6 years for single lung transplant patients.^14^, ^15^ Further, in excess of 34,000 LT recipients developed failure of the lung allograft between 1992 and 2017, and hence required either retransplantation or succumbed to a complication, most frequently CLAD.^14^ Although surveillance bronchoscopy with TBBx has been adopted as SOP for the majority of LT programs, this has never been scrutinized in randomized controlled trials designed to assess efficacy for this strategy.^16^ Additionally, SLT recipients often represent a higher risk demographic with potential intolerance for invasive TBBx procedures due to advanced age, comorbidity, and near-total dependence on unilateral allograft function. Therefore, a noninvasive surveillance solution implementing dd-cfDNA should represent a valuable adjunct in providing personalized patient care. Hitherto, only limited published data have specifically addressed experiences with dd-cfDNA in the SLT population.

The principal findings of our analysis were several. First, the dd-cfDNA fraction, when corrected by a CLIA-lab algorithm (multiplied by factor of 2) for use with the DLT-developed threshold of 1%, detected a spectrum of complications associated with allograft injury in SLT patients, including AR, INFXN, and CLAD. Second, dd-cfDNA testing among this SLT cohort had a performance similar to those that have been reported in double-lung transplant patients (sensitivity: 55.6%-100%, specificity: 73%-84%, PPV: 43%-64%, NPV: 83%-90%).^17^, ^18^, ^19^, ^20^ Indeed, Keller et al at the National Heart, Lung and Blood Institute (NHLBI) have recently reviewed their comprehensive data from 2 published prospective observational studies with dd-cfDNA by “*shotgun sequencing*” after LT and similarly concluded the appropriateness of a correction factor for SLT dd-cfDNA raw values. Their analysis, including 65 SLT and 156 DLT patients, found an uncorrected dd-cfDNA fraction for SLT vs DLT of 0.15% vs 0.46% (*p* < 0.01) in the healthy control cohort, 1.06% vs 1.78% (*p* = 0.05) in the AR cohort, and 0.64% vs 1.64% (*p* = 0.08) in the infection (with graft dysfunction) cohorts.^18^ The *corrected* SLT fractions in our study differed slightly from those of Keller and colleagues, most likely due to inherent differences in methodology, study design, and subject cohorts; nevertheless, these studies demonstrate consonant dd-cfDNA trends and clinical validity across diagnostic cohorts. Their AUROC for analysis of AR vs STABLE for SLT (0.89) and DLT (0.86) is also consonant with our finding (0.85). Further, the dd-cfDNA test performance for SLT in our study was robust for detection of AR with sensitivity = 77.8%, specificity = 84.6%, PPV = 38.3%, NPV = 96.8%, +LR = 5.0, and −LR = 0.26. The clinical value of dd-cfDNA for surveillance is evidenced by the high NPV and sensitivity for AR. Intriguing, however, is that the lower PPV may not necessarily be attributed to “false positive” results since dd-cfDNA elevation can predate the clinical or histopathologic AR event or relate to nonimmunologic causes of graft injury.^17^, ^21^ Further, Keller et al recently described a significant difference in hazard ratios for CLAD and death associated with histopathologic-confirmed ACR with an elevated dd-cfDNA fraction (≥1.0%) when compared to episodes of rejection with subthreshold dd-cfDNA (HR 3.13 vs 1.15).^22^ Therefore, dd-cfDNA clinical value may not only be limited to high NPV. If confirmed in additional large prospective studies, elevated levels in dd-cfDNA may also have prognostic implications. Additionally, as demonstrated in our “OTHER” cohort, other sources of graft injury aside from rejection and infection can be associated with elevation in dd-cfDNA fraction.

Second, although we noted an insignificant trend for higher dd-cfDNA fraction in left vs right side lung allografts during AR events (2.43% vs 1.58%, respectively), considering the lack of discernible dd-cfDNA differences in the STABLE and combined cohorts, the data suggest no significant dd-cfDNA differences based on laterality of SLT.

There are several limitations to our study. First and foremost, this was a CLIA-laboratory Quality Initiative with limitations inherent to a retrospective study design. Second, each dd-cfDNA test was assigned to an allograft status cohort based on clinical and histopathological data in a 6-week (42-day) window before dd-cfDNA testing, thus clinical events outside of that window that could have affected classifications may have gone unnoticed. This strategy was implemented based on published data from NHLBI (GRAfT Consortium) that dd-cfDNA perturbations occur a median of 2.8 months and 1 month before clinical detection of AMR or ACR, respectively.^17^, ^21^ Regardless, planned future analyses should attempt to more precisely temporally match the dd-cfDNA data with contemporaneous clinical and histopathologic events. Third, although the episodes of nonhistologically confirmed but clinically suspected AR were excluded from analysis, these nonetheless do represent the reality of clinical care and may be appropriate for inclusion in future studies with strictly defined criteria. Fourth, an in-depth analysis of CLAD was limited by the small sample size in our investigation. However, the focus of this report was principally on treatable “risk factors” for CLAD such as AR and infection since CLAD onset is defined by clinical criteria with decrement in forced expiratory volume (FEV)-1/sec and exclusionary diagnoses. In prospective multicenter studies already in progress, we intend to analyze longitudinal dd-cfDNA “kinetics” to potentially detect CLAD at a subclinical stage (i.e., before symptoms or spirometry decline). Finally, although the sample size for the AMR cohort was small, dd-cfDNA was shown to be significantly elevated in LT patients with either form of AR or graft injury; moreover, the cohort size of this study was impressive given the relatively small proportion of SLT among the total LT population.

This report represents the largest study to date with a multicenter design, which reports plasma dd-cfDNA test performance specifically in the vulnerable SLT population. Further, these real-world experiences support the clinical validity and utility of dd-cfDNA for monitoring of these patients. Looking forward, the impact of biomarker surveillance on clinically meaningful outcomes should be forthcoming from ongoing robust, prospective, clinical trials in progress. Additionally, attempts to enhance the predictive power of dd-cfDNA by combining plasma dd-cfDNA results with other biomarkers using unassisted machine learning algorithms are currently underway, with a focus on differentiating AR from INFXN.

## Author contributions

Conceptualization: D.J.R., S.B; Methodology: A.A., F.A., J.P.R., R.R., R.G., H.H., D.J.R.; Data Collection: A.A., F.A., J.P.R., R.R., R.G., H.H., D.J.R., K.C., S.M.C.; Formal analysis: D.J.R., Z.Z.; Resources: J.P.R., B.C.K.; Data curation: A.A., F.A., J.P.R., R.R., R.G., H.H., D.J.R.; Writing original draft: D.J.R.; Writing - reviewing and editing: A.A., F.A., J.P.R., R.R., R.G., H.H., K.C., S.M.C., Z.Z., S.B., D.J.R.; Supervision: D.J.R., S.B. All authors reviewed and approved of the final manuscript.

## Disclosure statement

The authors declare the following financial interests/personal relationships which may be considered as potential competing interests: David J. Ross, Sangeeta Bhorade, Sarah McCormick, Kathryn Crabtree, and Zhiji Zhang report a relationship with Natera, Inc. that includes employment and equity or stocks. Justin Rosenheck reports a relationship with Zambon Pharmaceutical Laboratories that includes consulting or advisory and speaking and lecture fees. Howard Huang reports a relationship with CareDx Inc. that includes consulting or advisory. The other authors declare that they have no known competing financial interests or personal relationships that could have appeared to influence the work reported in this paper.

This study received no funding.

## Footnotes

1 Prospera is a Natera-owned trademark.
