# Corrigendum to “Donor-derived cell-free DNA is a valuable monitoring tool after single lung transplantation: Multicenter analysis” [JHLT Open, 6 (2024) 100155]

**DOI:** 10.1016/j.jhlto.2024.100196

**Published:** 2025-01-07

**Authors:** Ambalavanan Arunachalam, Fatima Anjum, Justin P. Rosenheck, Reinaldo Rampolla, Reda Girgis, Howard J. Huang, Kathryn Crabtree, Sarah McCormick, Zhiji Zhang, Sangeeta Bhorade, David J. Ross

**Affiliations:** a Division of Pulmonary and Critical Care, Northwestern University Feinberg School of Medicine, Chicago, Illinois; b Lewis Katz School of Medicine, Temple University, Philadelphia, Pennsylvania; c Division of Pulmonary, Critical Care, and Sleep Medicine, The Ohio State University, Columbus, Ohio; d Cedars-Sinai Medical Center, Department of Cardiac Surgery, Los Angeles, California; e Corewell HealthCare and Michigan State University College of Human Medicine, Grand Rapids, Michigan; f Houston Methodist Lung Transplant Center, Houston Methodist Hospital, Houston, Texas; g Natera, Inc., Austin, Texas

The authors regret that they did not include the full range of times, from the date of single lung transplantation until the initial dd-cfDNA lab draw. For all cohorts combined, the median time was 233 days (IQR: 96-489; range: 27-7,795) days.

The authors would like to apologize for any inconvenience caused.

